# Soil microbial community responses to active and passive restoration of selectively logged Bornean tropical forest

**DOI:** 10.3389/fmicb.2025.1570294

**Published:** 2025-08-22

**Authors:** Samuel J. B. Robinson, Dafydd M. O. Elias, Tim Goodall, Niall P. McNamara, Robert Griffiths, Noreen Majalap, Yap Sau Wai, Nicholas J. Ostle

**Affiliations:** ^1^UK Centre for Ecology and Hydrology, Lancaster, United Kingdom; ^2^Lancaster Environment Centre, Lancaster University, Lancaster, United Kingdom; ^3^UK Centre for Ecology and Hydrology, Wallingford, United Kingdom; ^4^School of Natural Sciences, Bangor University, Bangor, United Kingdom; ^5^Sabah Forestry Department, Forest Research Centre, Sandakan, Malaysia; ^6^Faculty of Tropical Forestry, Universiti Malaysia Sabah, Kota Kinabalu, Malaysia

**Keywords:** 16S, ITS, ectomycorrhizal fungi, forest rehabilitation, liana cutting, climber cutting, liberation thinning, distance decay

## Abstract

Tropical rainforests support critical biogeochemical cycles regulated by complex plant-soil microbial interactions but are threatened by global change. Much of the uniquely biodiverse and carbon rich forest on Borneo has been lost through extensive conversion to monoculture plantation, and a significant proportion of the remaining forest has been heavily modified by selective logging. Ecological restoration of tropical forest aims to return forests to a near pristine state, but restoration initiatives are hindered by limited understanding of the underpinning plant-soil feedbacks, and impacts on soil microbial communities are unresolved. We characterized soil properties and soil bacterial and fungal communities using amplicon sequencing across adjacent old-growth and selectively logged lowland dipterocarp forest in Borneo undergoing either natural regeneration or restoration by enrichment planting. While many soil properties were similar across forest types, we found contrasting responses of different soil microbial groups to active and passive restoration. Bacterial and fungal community composition were generally distinct in old-growth forest and more similar in logged forest. Bacterial alpha diversity and rate of spatial turnover appeared to recover toward old-growth forest with active restoration, while fungal alpha diversity showed slower signs of recovery. The composition and rate of spatial turnover in mycorrhizal communities was most different between old-growth and actively restored forest, possibly resulting from mycorrhizal associations of tree species planted during restoration. Surprisingly, old-growth forest shared fewer microbial taxa with actively restored forest than with naturally regenerating forest, suggesting current restoration practices (removal of lianas and understorey vegetation) may be selecting for different microbial communities. Taken together, our findings show that certain attributes of key soil microbial groups remain distinct from old-growth forest almost two decades after logging disturbance, and some may diverge with active restoration. Changes in enrichment planting practices to promote rehabilitation of belowground communities may be required for successful biodiversity conservation and recovery of vital ecosystem functions.

## 1 Introduction

Old-growth forests are rapidly being replaced by human-modified secondary forest worldwide, with highest conversion rates in the tropics (Keenan et al., 2015), affecting the crucial role of tropical forests as global reservoirs of biodiversity and carbon (C) (Baccini et al., 2017; Myers et al., 2000; Pan et al., 2011; Powers and Jetz, 2019; Qie et al., 2017). The forests of Borneo are a hotspot of forest disturbance and loss, driven by timber extraction and conversion to oil palm plantation. A reduction of forest cover by more than 30% since the early 1970s means increasing pressure on the remaining forest to provide vital ecosystem services. However, almost half (16.8 Mha) of the remaining forest has been heavily modified through selective logging for the commercially valuable and canopy-dominant dipterocarp trees (Appanah and Turnbull, 1998; Gaveau et al., 2014; Gaveau et al., 2016). Their targeted removal and creation of landings and skid trails affects forest structure, plant and soil microbial communities, ecosystem C cycling and C sequestration (Asner et al., 2018; Both et al., 2019; Ellis et al., 2016; Marsh et al., 2025; Riutta et al., 2018; Robinson et al., 2020; 2024). This practice reflects regional and global trends, as selective logging is widespread across Southeast Asia (Stibig et al., 2014) and the primary driver of tropical forest modification, affecting more than half of tropical forest worldwide (Potapov et al., 2017; Potapov et al., 2008).

Forest disturbance can significantly affect soil physicochemical properties, often reducing soil C pools (Don et al., 2011; Wei et al., 2014), altering nutrient availability and overall fertility (Daljit Singh et al., 2013; Paul et al., 2010). Changes in soil properties can impact on soil microbial communities (Jesus et al., 2009; Tripathi et al., 2012) by determining availability of effective resources and creation of different ecological niches (Zhang et al., 2018). Soil microbial diversity may increase with a certain level of disturbance (Ferrenberg et al., 2013; Galand et al., 2016; Wilkinson, 1999; Zhang et al., 2011), while changes in community structure and function may directly relate to disturbance intensity (Berga et al., 2012). Soil fungal communities, particularly mycorrhizae, have been shown to be highly sensitive to logging disturbance, likely as dipterocarp trees are an ectomycorrhizal-associating species (Kerfahi et al., 2014; McGuire et al., 2015; Robinson et al., 2020; Robinson et al., 2024). Logging effects on soil bacterial communities are less clear; two studies in Malaysian Borneo found no impact of logging on either local bacterial alpha (Shannon) diversity or beta diversity (i.e., spatial heterogeneity in bacterial communities, evaluated using dispersion of community dissimilarities) (Lee-Cruz et al., 2013; Tripathi et al., 2016), while other studies revealed shifts in bacterial community composition between intact forest and selective logging gaps (Robinson et al., 2024) and effects of logging on change in bacterial community composition down the soil profile (Tin et al., 2018). This also highlights the importance of fine spatial resolution in assessment of soil bacterial communities which may vary at the meter- or even centimeter-scale (O’ Brien et al., 2016). Disturbance impacts on soil microbial communities may have consequences for biogeochemical cycling that is regulated by complex reciprocal feedbacks between plants and soil (Bever et al., 2010; Cortois et al., 2016; van der Heijden et al., 2008; van der Putten et al., 2013; Wardle et al., 2004). For example, a reduction in cycling rates of certain key soil nutrients has been associated with selective logging canopy gaps, with soil heterotrophic respiration negatively related to disturbance intensity (Robinson et al., 2024).

Although the preservation of primary forest is crucial for biodiversity conservation and the maintenance of ecosystem services (Gibson et al., 2011), natural and managed restoration of secondary tropical forest has great potential for the recovery of ecosystem functions including C storage (Chazdon, 2008; Melo et al., 2013; Pan et al., 2011; Philipson et al., 2020; Wright, 2010). Rehabilitation of disturbed tropical forest via planting programmes is now a widely used strategy to recover vegetation structure and diversity (Bonner et al., 2019; Celis and Jose, 2011; Shoo et al., 2016). Enrichment planting, the reintroduction of native tree species removed through human disturbance, has been adopted across Southeast Asia as a strategy to restore forest floristic composition toward that of old-growth forest (Axelsson et al., 2024; Banin et al., 2023; Perumal et al., 2016). This practice is often accompanied by liana and climber removal, liberation cutting and clearing of understorey vegetation along planting lines, to facilitate seedling establishment by reducing competition for resources. This approach is currently employed in large-scale restoration projects, for example over the 25,000 ha Ulu-Segama area in the Malaysian state of Sabah where forest degradation has been most extreme (Axelsson et al., 2024; Bartholomew et al., 2024; CIFOR-ICRAF, 2024; Face the Future, 2025; Gaveau et al., 2014; Hayward et al., 2021; Reynolds et al., 2011). However, success of tropical forest planting programmes is generally limited by lack of context-specific knowledge of the ecology of planted tree species, including plant-soil interactions (e.g., Rodrigues et al., 2009). Most enrichment planting studies in Southeast Asian tropical forest have focused on tree survival, growth and productivity, with less consideration of soil biodiversity and functioning (Banin et al., 2023; Perumal et al., 2016; Veryard et al., 2023). Following replanting of clear-felled forest, some studies observed increases in microbial biomass carbon (MBC) (Deng et al., 2010; Nurulita et al., 2016) and bacterial alpha diversity accompanied by bacterial community shifts that may indicate ecosystem recovery (Deng et al., 2010). The small number of studies undertaken in enrichment planted secondary Malaysian forest highlight increases in microbial biomass toward that of old-growth forest (Daisuke et al., 2013; Daljit Singh et al., 2013; Perumal et al., 2016). However, microbial indicators can be system-specific and depend on type, intensity and duration of disturbance (Banning et al., 2011). Understanding of the capacity to restore soil microbial diversity, physicochemical properties and biogeochemical cycling in hyper-diverse tropical forest is broadly limited (Bonner et al., 2019), reflecting a general lack of understanding of the patterns of microbial responses to ecosystem restoration (Banning et al., 2011; Strickland et al., 2017).

To address these knowledge gaps, we surveyed soil microbial community attributes, soil physicochemical properties and forest structural characteristics across old-growth and selectively logged forest undergoing either passive natural regeneration or active restoration by enrichment planting. We tested the following specific hypotheses:

1. Soil microbial communities (composition and diversity) will differ across forest types, with greater similarity between old-growth and actively restored forest.
2. Microbial biomass will increase with active restoration toward old-growth forest.
3. Soil physicochemical properties, microclimate and forest structural characteristics will differ across forest types, corresponding to expected differences in soil microbial community attributes.

## 2 Materials and methods

### 2.1 Study sites

**FIGURE 1:**
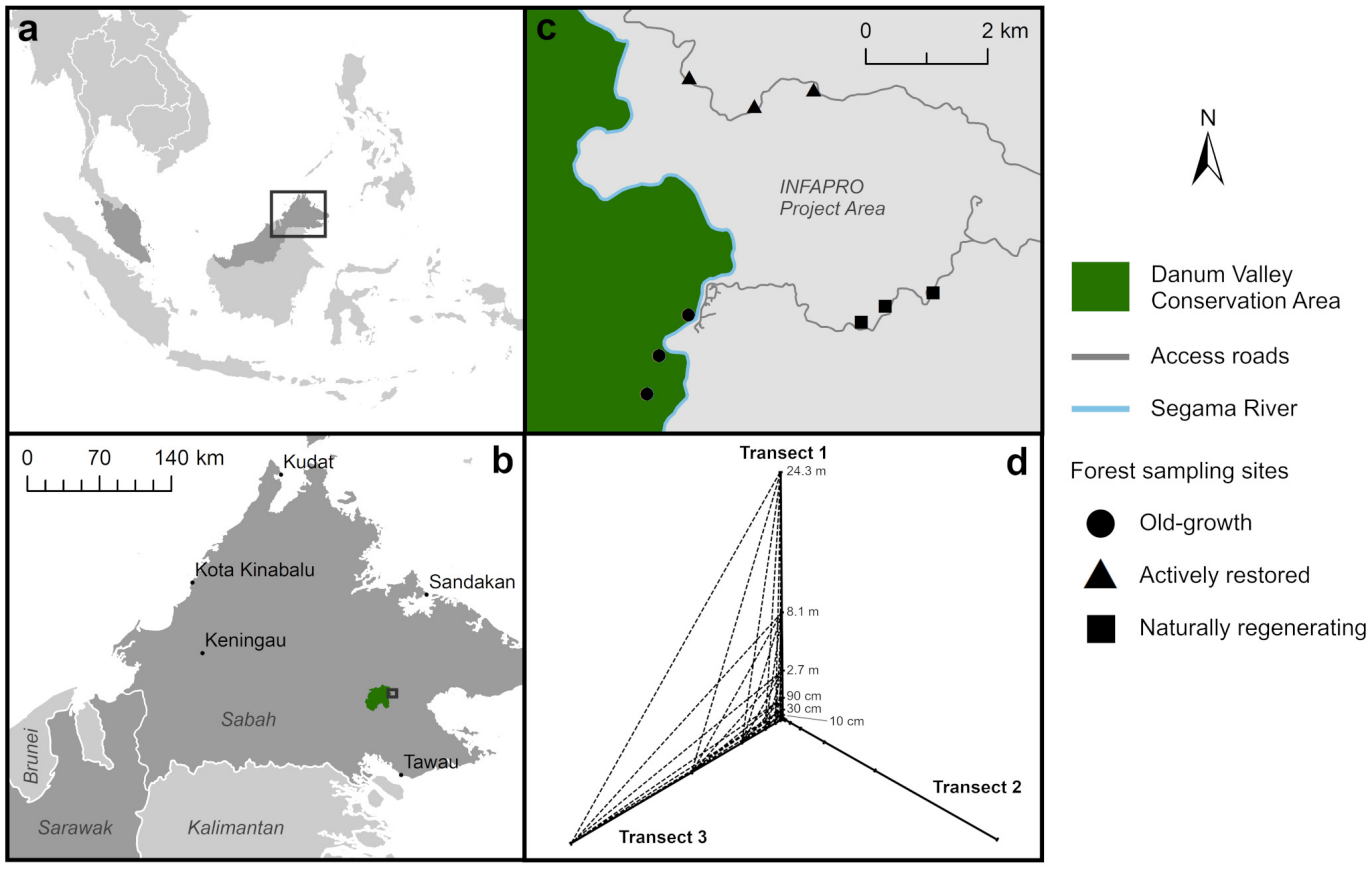
Map of sampling sites in northern Malaysian Borneo **(a)** in the state of Sabah **(b)**, located within old-growth forest (Danum Valley Conservation Area), and adjacent naturally regenerating and actively restored selectively-logged forest (INFAPRO forest rehabilitation project) **(c)**. At each site, soil was collected following a geospatial sampling design **(d)**, at increasing distances along three transects radiating out from one central point. Dotted lines illustrate example distances between sampling points of different transects (here between transects 1 and 3) used to calculate distance decay indices for evaluation of spatial turnover of soil microbial taxa, in addition to distances along each respective transect.

This study was conducted in the state of Sabah, northern Malaysian Borneo. The climate is moist tropical (annual precipitation 2,600–2,700 mm, average daily temperature 27°C) and generally non-seasonal, but may undergo irregular inter-annual dry periods averaging ∼1.4 months of the year (Kumagai and Porporato, 2012; Walsh and Newbery, 1999). Sampling was conducted in March 2018 at nine sites distributed across old-growth and naturally regenerating or actively restored selectively logged lowland dipterocarp rainforest (3 sampling sites in each) (Figure 1). Logged forest sites were situated within the Innoprise Face Foundation Rainforest Rehabilitation Project (INFAPRO) area (4.99°N, 117.86°E). This large-scale restoration initiative, in partnership with the Yayasan Sabah Foundation, aims to restore 25,000 ha within the Ulu-Segama forest management unit (Face the Future, 2025). All logged forest sites were situated within the same logging coupe that was selectively logged once in 1989. The actively restored forest has undergone rehabilitation by enrichment planting since 2000, with mixtures of dipterocarp species, non-dipterocarp canopy-forming species and various fruit tree species planted at 3 m intervals along 10 m spaced parallel transects. Planting lines were maintained by regular liana cutting and removal of competing understorey vegetation for the initial 3 years following enrichment planting (Bartholomew et al., 2024; CIFOR-ICRAF, 2024; Hayward et al., 2021; Moura Costa, 1996). Old-growth forest was located in the adjacent Danum Valley Conservation Area (DVCA) (4.95°N, 117.79°E), a 438 km^2^ rainforest reserve that has undergone little or no anthropogenic disturbance having been legally protected from commercial timer operations since 1976 (Marsh and Greer, 1992). Recent extensive tree community surveys across the Ulu-Segama Forest Reserve (including the coupe sampled in this study) and adjacent DVCA have shown persistent shifts in tree species composition and reduced basal area 23–35 years after logging disturbance, regardless of restoration method (Hayward et al., 2021), with corresponding negative impacts on recruitment and diversity of tree seedlings (Bartholomew et al., 2024).

### 2.2 Sampling design for soil, microclimate and forest structural characteristics

Nine sampling sites were distributed across a contiguous area of forest that had undergone contrasting logging disturbance and restoration. Three sampling sites were chosen in each of three forest types of old-growth, naturally regenerating and actively restored logged forest, situated between a minimum of 500 m and maximum of 5.7 km apart. At each site, a geospatial transect design was used for soil sampling and measurement of microclimate and forest structural characteristics. This comprised three transects radiating out from one center point positioned at 120° to one another, with the first transect oriented North (Figure 1d). Soil and microclimate sampling points were located at the center point, then at six points along each of the three transects at increasing distances of 10 cm, 30 cm, 90 cm, 2.7 m, 8.4 m and 24.3 m relative to the center. This resulted in nineteen soil samples per site, totaling 171 across all sites, which were analysed individually. Soil cores were collected at each sampling point a using a 3 cm diameter gouge auger to a depth of approximately 10 cm for analysis of soil microbial community attributes and physicochemical analysis. The organic soil layer was collected by separating from underlying mineral soil and sealing in a Ziploc bag, and was then transported to a laboratory. Multiple cores were taken at each sampling point to ensure enough material for biological and physicochemical analyses due to the shallow organic layer depth. Samples were hand-homogenized and ∼10 g subsamples taken for analysis of soil microbial community attributes. These were frozen at −20°C on the day of collection and transported on ice to the United Kingdom for analysis of soil microbial communities: 5 g was transported to the United Kingdom Centre for Ecology and Hydrology, Wallingford for amplicon sequencing and 5 g to Lancaster University for phospholipid fatty acid (PLFA) analysis. The remaining soil was transported to the Sabah Forest Research Centre, Sepilok for physicochemical analysis.

Microclimate variables were measured on the day of soil sample collection. Soil temperature (approximate depth 0–10 cm) and understory air temperature at the soil surface (5–15 cm) were measured with using a thermistor (Salter, United Kingdom). Understory photosynthetically active radiation (PAR) was measured using a light meter (PP Systems, United States) with the sensor held just above the soil surface.

Forest structure was evaluated by recording and measuring circumferences of all stems with diameter at breast height (DBH) >5 cm within a 2.5 m buffer of all transects, for calculation of stem density and basal area. This DBH was chosen to capture finer-scale variation in tree abundance. Stem density and basal area were calculated for each sampling location (*n* = 9).

### 2.3 Soil physicochemical analysis

pH in water was measured on fresh soils using a pH meter with a combination glass-calomel electrode (a ratio of 1:2.5 soil to deionized water) after shaking overnight at 100 rev min^–1^ on an orbital shaker and standing for 30 min (Landon, 1984). The remaining soils were air-dried at 40°C to constant weight and passed through a 2 mm sieve for homogenization and removal of roots and stones. Subsamples for total C and N analysis were dried at 65°C for 48 h and milled to a fine powder with a pestle and mortar. Total soil C and N contents were determined by dry combustion at 900°C using an Elementar Vario Max CN analyser (Elementar Analysensysteme, Hanau, Germany). For soil total P, samples were digested using sulphuric acid-hydrogen peroxide (Allen, 1989). Inorganic P was extracted using a Bray No. 1 extractant (Bray and Kurtz, 1945). P contents of extracts and digests were determined using the molybdenum-blue method (Anderson and Ingram, 1993), read at 880 nm on a spectrophotometer (HITACHI-UV-VIS, Japan).

### 2.4 Soil phospholipid fatty acid (PLFA) analysis

A subset of soil samples were analysed for PLFAs to provide indicators of total microbial biomass and relative abundances of bacteria and fungi across forest types. Due to the intensive extraction requirements, 36 of the 171 samples were analysed (four samples per sampling location). These corresponded in each site to the transect center point and 30 cm, 2.7 m and 24.3 m sampling points along the first transect arm. PLFAs were extracted from 1.8 g freeze dried soil after removal of coarse roots and stones using a modified Bligh and Dyer extraction method (White et al., 1979). Extracts were analysed using an Agilent 6890 Gas Chromatograph with Flame Ionization Detector (GC-FID; Agilent Technologies, Unites States) using an RTx-1 capillary column (60 m × 0.32 mm ID, 0.25 μm film thickness). PLFA peaks were identified using retention times calibrated against known standards. As indicators of Gram-positive bacterial biomass, the branched-chained fatty acids C15:0i, C15:0a, C16:0i, 7Me-C17:0, C17:0i and C17:0a were used (Haack et al., 1994; Lechevalier and Lechevalier, 1988; O’Leary and Wilkinson, 1988; Rinnan and Bååth, 2009; Whitaker et al., 2014; Zelles, 1999). For Gram-negative bacteria, the monounsaturated fatty acids C16:1ω7c, C16:1ω5, C18:1ω7c and cyclopropane fatty acids cyC17:0 and cyC19:0 were used (Rinnan and Bååth, 2009; Whitaker et al., 2014; Zelles, 1999). For fungi, the fatty acids C18:2ω6,9 and C18:1ω9 were used (Bååth and Anderson, 2003; de Deyn et al., 2011). Total bacterial biomass was calculated as the sum of Gram-positive and Gram-negative PLFAs and the fatty acid C15:0 (de Deyn et al., 2011). Fungal to bacterial ratio (F:B) was calculated as the proportion of total bacterial relative to total fungal PLFAs. Total microbial PLFAs were determined as the sum of all identified PLFAs, including those above and the additional fatty acids C14:0, C16:1, C16:0, C17:1ω8, C17:0br, C18:0br, C18:1ω5, C18:0 and C19:1. PLFA contents were expressed as μg g^–1^ dry soil. Four samples (two samples from old-growth and two from naturally regenerating logged forest, all from different sites) were omitted from analysis due to extraction error, resulting in a total of 32 PLFA samples.

### 2.5 Molecular analysis of soil microbial communities and data pre-processing

Molecular analyses and bioinformatics were conducted using all soil samples collected (*n* = 171; 57 per forest type) following methods described by Robinson et al. (2024; 2020). DNA was extracted from 0.2 g soil using the PowerSoil^®^ DNA Isolation Kit and protocol (MoBio Laboratories). Amplicon libraries were constructed according to a dual indexing strategy with each primer consisting of the appropriate Illumina adapter, 8-nt index sequence, a 10-nt pad sequence, a 2-nt linker and the amplicon specific primer (Kozich et al., 2013). Bacteria were targeted using V3-V4 16S rRNA amplicon primers CCTACGGGAGGCAGCAG and GCTATTGGAGCTGGAATTAC (Kozich et al., 2019). For fungi, the ITS2 region was amplified using primers fITS7 GTGARTCATCGAATCTTTG (Ihrmark et al., 2012) and ITS4 TCCTCCGCTTATTGATATGC (White et al., 1990). Although the capability of detecting AM fungi using ITS primers is debated (Hart et al., 2015), recent studies have shown that patterns in diversity and community composition can be adequately identified within sample types such as soil (Berruti et al., 2017; Lekberg et al., 2018). Amplicons were generated using a high-fidelity DNA polymerase (Q5 Taq, New England Biolabs). After an initial denaturation at 95°C for 2 min, PCR conditions were as follows: Denaturation at 95°C for 15 s; annealing at 55°C (bacteria) 52°C (fungi); annealing times were 30 s with extension at 72°C for 30 s; cycle numbers were 25 for bacteria and fungi; a final extension of 10 min at 72°C was included. Amplicon sizes were determined using an Agilent 2200 TapeStation system, samples were normalized using SequalPrep Normalization Plate Kit (Thermo Fisher Scientific) and pooled. The pooled library was quantified using a Qubit dsDNA HS kit (Thermo Fisher Scientific) prior to sequencing with an Illumina MiSeq using V3 600 cycle reagents at a concentration of 8 pM with a 5% PhiX Illumina control library. Sequences were processed in R using DADA2 to quality filter, merge, de-noise and assign taxonomies (Callahan et al., 2016). Forward sequence reads were used for 16S (trimmed to 250 bases), while forward and reverse were used for ITS (trimmed to 225 and 160 bases, respectively). Filtering settings were maximum number of Ns (maxN) = 0, maximum number of expected errors (maxEE) = 1. Sequences were dereplicated and the DADA2 core sequence variant inference algorithm applied. mergePairs and remove BimeraDenovo functions were used at default settings to merge ITS forward and reverse reads and remove chimeric sequences. The amplicon sequence variants (ASVs) were subject to taxonomic assignment using assignTaxonomy with default bootstrapping (50) and the training database UNITE version 7.2 (Abarenkov et al., 2010).

Fungal functional guild classifications were assigned to ASVs using the FUNGuild annotation tool (Nguyen et al., 2016). Only ASVs with unambiguous (non-multiple) classifications of “probable” or “highly-probable” confidence rankings were considered for analysis. These were used for calculating relative abundances of fungal guilds and sub-setting saprotrophic, mycorrhizal, ectomycorrhizal and pathogenic fungal datasets for assessment of diversity and community dissimilarity.

Sequencing data were pre-processed and alpha diversity indices (ASV richness, Shannon index) and fungal guild relative abundances calculated in R (R Core Team, 2025) using the *phyloseq* package (McMurdie and Holmes, 2013). PCRs failed for three bacterial samples (two from different naturally regenerating logged forest sites, one from actively restored logged forest) as indicated by abnormally low read counts, and these samples were excluded from all subsequent analyses. Only ASVs assigned to the kingdoms of Bacteria or Fungi were retained for downstream analysis, and all singleton ASVs were removed. Sub-setting by fungal guilds was conducted on the full unrarefied dataset to maximize the number of ASV reads available for analysis of functional groups. Sample sequencing depth was normalized for each group by rarefying to the minimum read counts per sample for bacterial (3,778 reads), and total fungal (3,868), saprotrophic (472), mycorrhizal (20), ectomycorrhizal (6) and pathogenic (69) fungal groups.

### 2.6 Statistical analyses

All statistical analyses were conducted in R (R Core Team, 2025), and significance of all tests was considered at the *p* ≤ 0.05 level. To test the differences in univariate soil microbial community attributes (alpha diversity metrics and fungal guild relative abundances) and soil, microclimate and forest structural characteristics between forest types, linear mixed effects regression models (LMMs) were constructed in the *lme4* R package (Bates et al., 2015) with site ID included as a random intercept term to control for within-site pseudoreplication. Pairwise comparisons between forest types were conducted with the *emmeans* R package with Bonferroni correction (Lenth et al., 2019). Normality of model residuals were evaluated using Shapiro-Wilk tests and Q-Q plots, and variables were log-, square root- or exp- transformed where necessary to improve model fit.

Soil microbial community data were Hellinger-transformed prior to analysis (Legendre and Borcard, 2018) to control for the effect of rare taxa and merged at the site level (*n* = 9) to control for spatial pseudoreplication. Soil microbial community compositions across forest types were visualized with PCoA using Bray-Curtis dissimilarities. Differences in soil microbial community composition between forest types were tested with PERMANOVA in vegan (Oksanen et al., 2019) and homogeneity of multivariate dispersion between forest types was evaluated. All permutational tests were run with 9,999 permutations. Pairwise comparisons of soil microbial community dissimilarities between forest types could not be carried out due to the low number of true replicates, restricting the number of possible permutations for calculating significance level. UPGMA (unweighted pair-group method with mathematic average) hierarchical cluster analysis was performed using the hclust R function (R Core Team, 2025) to identify groups of more (dis)similar sites across forest types.

We employed a distance-decay approach to assess the response of microbial beta diversity to forest management, a biogeographical method widely used to study spatial turnover in macro-organism communities that is now being applied to soil microbes due to advances in molecular techniques (Green et al., 2004; Martiny et al., 2006; Morlon et al., 2008; Nekola and White, 1999). Distance-decay relationships can be derived using geographic distances from centimeters to kilometers simultaneously (e.g., Barreto et al., 2014). This allowed us to evaluate microbial community turnover with environmental heterogeneity across scales not previously studied in old-growth and disturbed tropical forest, and to address unresolved disturbance effects on soil microbial diversity that may be spatially dependent. Pairwise Bray-Curtis community dissimilarities for soil microbial groups and corresponding geographic distances were calculated between the 19 sampling points in each sampling location. This provided a total of 171 pairs per location, with geographic distances ranging from 10 cm to 42.09 m. Linear regression was used to obtain the coefficient of the distance decay relationship (*Y*), i.e., rate of spatial turnover in soil microbial taxa, between log-transformed Bray-Curtis dissimilarities and log-transformed geographic distances for each location and soil microbial group (Barreto et al., 2014; Nekola and White, 1999). ANOVA with Tukey HSD *post hoc* tests were used to test differences in Y-values between forest types (*n* = 9).

Numbers of shared and distinct soil microbial ASVs between forest types were visualized with Venn diagrams using the *RAM* R package (Chen et al., 2018). Indicator analysis was conducted to identify specific soil microbial taxa uniquely associated with different forest types using the mulitpatt function in the *indicspecies* R package (de Cáceres and Legendre, 2009). This provided an index of association (Indicator Value) between forest type and soil microbial ASVs and *p*-values denoting significant indicator taxa.

## 3 Results

### 3.1 Soil microbial diversity, community composition and biomass across old-growth, naturally regenerating and actively restored logged forest

**FIGURE 2:**
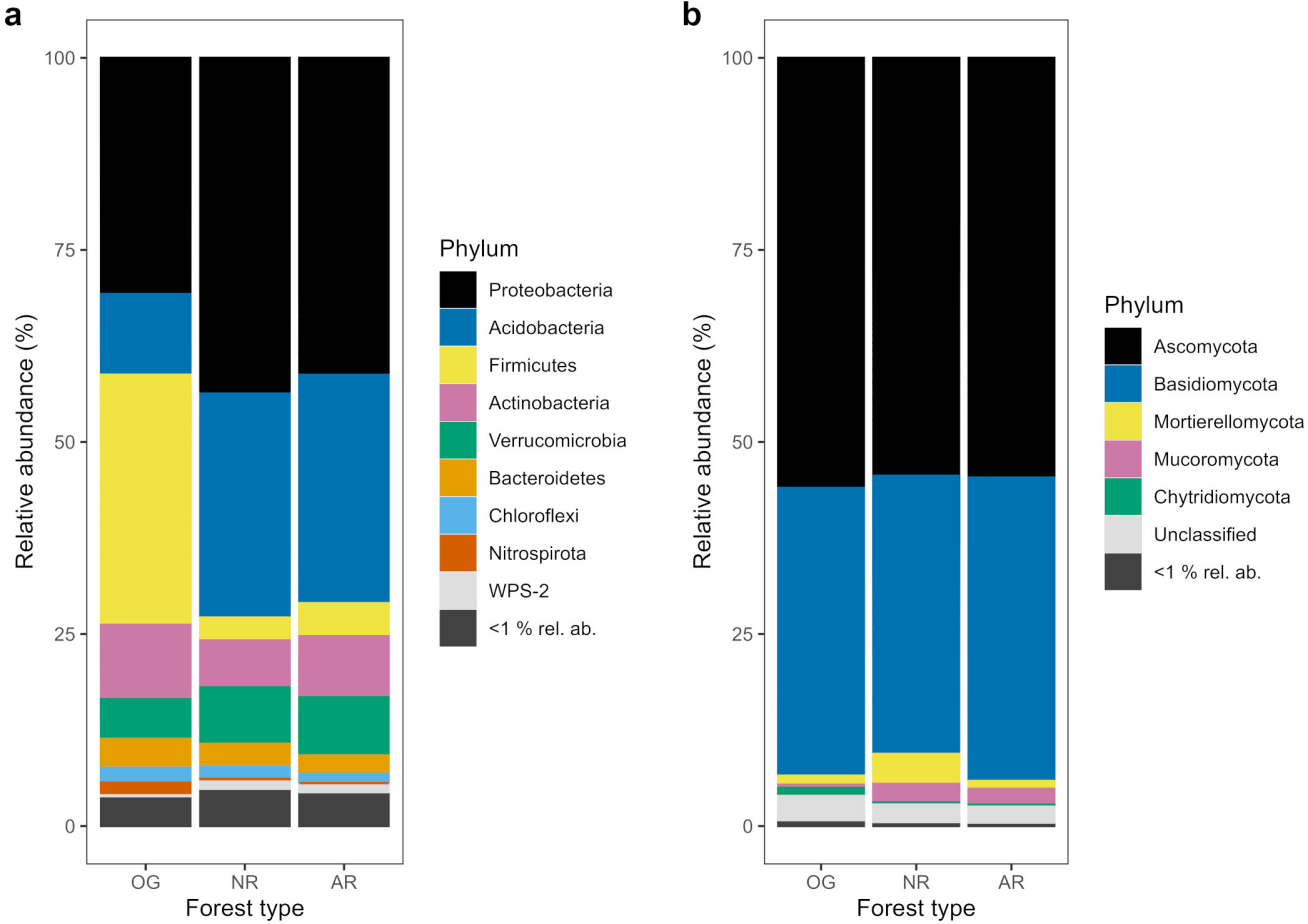
Relative abundances of bacterial **(a)** and fungal **(b)** phyla as percentages of total amplicon sequence variants (ASVs) across old-growth forest (OG), naturally regenerating logged forest (NR) and actively restored logged forest (AR) (*n* = 168 and *n* = 171 for bacterial and fungal phyla, respectively). Phyla with <1% relative abundance (rel. ab.) across all forest types are represented as one group.

**FIGURE 3:**
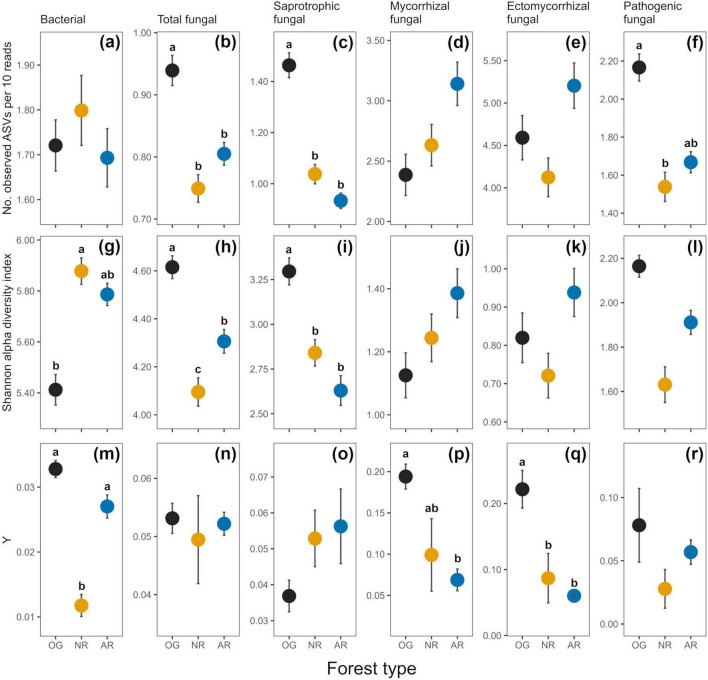
Indices of soil microbial alpha diversity [richness as no. observed ASVs per 10 reads **(a–f)**, and Shannon index **(g–l)**) and rate of spatial turnover of taxa (distance decay index; *Y* **(m–r)**] derived from amplicon sequence variants (ASVs) across old-growth forest (OG; black), naturally regenerating logged forest (NR; orange) and actively restored logged forest (AR; blue). Values are means ± 1 standard error. Lower case letters indicate statistically different or similar groups at the *p* < 0.05 level identified in *post hoc* tests after linear mixed model analysis. Sample numbers analysed were *n* = 168 for bacterial richness and Shannon diversity, *n* = 171 for fungal richness, Shannon diversity and fungal guild relative abundances, and *n* = 9 for both bacterial and fungal distance decay indices. See Table 2 for a summary of statistical test results.

In total, 37,379 bacterial ASVs (representing 44 identified phyla; 749 genera) and 21,298 fungal ASVs (12 phyla; 611 genera) were detected across all forest types (Figure 2 and Supplementary Figures 1, 2). In all soil microbial groups, a greater number of ASVs were only found in old-growth forest (14,000 bacterial, 7,695 fungal ASVs) compared to naturally regenerating (9,479 bacterial, 5,247 fungal) and actively restored logged forest (8,321 bacterial, 5,323 fungal), and generally more ASVs were shared between old-growth and naturally regenerating forest than between old-growth and actively restored forest (Supplementary Figure 3). Differences in alpha diversity indices or rate of spatial turnover of taxa (or both) were detected between forest types for all microbial groups studied (Figure 3). Mean bacterial Shannon alpha diversity was significantly higher in naturally regenerating forest relative to old-growth, while actively restored forest was similar to both (Figure 3g and Tables 1, 2). Total fungal richness and Shannon alpha diversity were significantly higher in old-growth relative to naturally regenerating and actively restored forest, while total fungal Shannon alpha diversity was higher in actively restored relative to naturally regenerating forest (Figures 3b, h). Saprotrophic fungal richness and Shannon alpha diversity were significantly higher in old-growth compared to both logged forest types (Figures 3c, i). Pathogenic fungal richness was higher in old-growth compared to naturally regenerating forest, while actively restored forest was similar to both (Figure 3f). Rates of spatial turnover of bacterial, total mycorrhizal fungal and ectomycorrhizal fungal taxa (distance decay index) differed by forest type. For bacteria, significantly lower *Y*-values (indicating slower spatial turnover) were found in naturally regenerating logged forest compared to old-growth and actively restored forest, which were similar (Figure 3m). Slower spatial turnover of both total mycorrhizal and ectomycorrhizal fungi was found in actively restored forest relative to old-growth forest. In naturally regenerating forest, spatial turnover of total mycorrhizal fungal taxa was similar to both other forest types, while spatial turnover of ectomycorrhizal fungal taxa was slower than old-growth and similar to actively restored forest (Figures 3p, q).

**TABLE 1:** Soil microbial community attributes in old-growth forest, naturally regenerating logged forest and actively restored logged forest (means ± 1 standard deviation). Superscript letters indicate statistically different or similar groups at the *p* < 0.05 level identified in *post hoc* tests after linear mixed model or Kruskal-Wallis analysis. Sample numbers analysed were *n* = 32 for Total PLFAs and Fungal: bacterial ratios, *n* = 168 for bacterial richness and Shannon diversity, *n* = 171 for fungal richness, Shannon diversity and fungal guild relative abundances, and *n* = 9 for both bacterial and fungal distance decay indices.

Parameter	Soil microbial group	Forest type		
		Old-growth	Naturally regenerating	Actively restored
Total PLFAs (μg g^–1^ dry soil)	Total microbial	51.27 ± 21.85	52.85 ± 17.58	59.13 ± 14.91
	Bacterial	26.56 ± 11.88	27.05 ± 8.90	30.19 ± 7.55
	Fungal	2.50 ± 0.64	3.42 ± 1.25	3.51 ± 1.11
Fungal: bacterial ratio	-	0.11 ± 0.04	0.13 ± 0.04	0.12 ± 0.02
Richness (no. observed ASVs 10 reads^–1^)	Bacteria	1.72 ± 0.43	1.80 ± 0.58	1.69 ± 0.49
	Total fungi	0.94 ± 0.18^a^	0.75 ± 0.17^b^	0.80 ± 0.14^b^
	Saprotrophic fungi	1.46 ± 0.37^a^	1.04 ± 0.29^b^	0.93 ± 0.23^b^
	Mycorrhizal fungi	2.39 ± 1.29	2.63 ± 1.29	3.14 ± 1.36
	Ectomycorrhizal fungi	4.59 ± 1.97	4.12 ± 1.73	5.20 ± 2.02
	Pathogenic fungi	2.17 ± 0.54^a^	1.54 ± 0.58^b^	1.67 ± 0.42^ab^
Shannon alpha diversity index	Bacteria	5.41 ± 0.45^b^	5.88 ± 0.38^a^	5.79 ± 0.33^ab^
	Total fungi	4.62 ± 0.36^a^	4.10 ± 0.45^c^	4.31 ± 0.37^b^
	Saprotrophic fungi	3.30 ± 0.57^a^	2.84 ± 0.56^b^	2.63 ± 0.63^b^
	Mycorrhizal fungi	1.13 ± 0.54	1.24 ± 0.57	1.39 ± 0.58
	Ectomycorrhizal fungi	0.82 ± 0.49	0.72 ± 0.44	0.94 ± 0.47
	Pathogenic fungi	2.16 ± 0.37	1.63 ± 0.61	1.91 ± 0.41
Distance decay index (rate of spatial turnover of taxa)	Bacteria	0.033 ± 0.002^a^	0.012 ± 0.003^b^	0.027 ± 0.003^a^
	Total fungi	0.053 ± 0.004	0.049 ± 0.013	0.052 ± 0.003
	Saprotrophic fungi	0.037 ± 0.008	0.053 ± 0.014	0.056 ± 0.018
	Mycorrhizal fungi	0.194 ± 0.026^a^	0.099 ± 0.076^ab^	0.069 ± 0.023^b^
	Ectomycorrhizal fungi	0.222 ± 0.049^a^	0.087 ± 0.065^b^	0.060 ± 0.004^b^
	Pathogenic fungi	0.078 ± 0.050	0.028 ± 0.026	0.057 ± 0.017
Fungal guild relative abundance (% total fungal ASV reads)	Saprotrophic fungi	44.73 ± 16.13	54.05 ± 24.82	42.72 ± 21.30
	Total mycorrhizal fungi	30.96 ± 18.21	31.70 ± 25.59	47.24 ± 24.97
	EcM fungi	30.21 ± 18.39	29.95 ± 26.62	45.98 ± 25.58
	AM fungi	0.66 ± 1.28	1.63 ± 2.41	1.11 ± 1.12
	Ericoid mycorrhizal fungi	0.09 ± 0.41	0.10 ± 0.19	0.14 ± 0.31
	Orchid mycorrhizal fungi	0.00 ± 0.01	0.01 ± 0.10	0.00 ± 0.02
	Total pathogenic fungi	17.22 ± 8.17^a^	11.51 ± 6.89^a^	7.27 ± 4.18^b^
	Plant pathogenic fungi	10.93 ± 6.06^a^	4.56 ± 5.06^b^	4.21 ± 3.42^b^
	Animal pathogenic fungi	6.29 ± 4.71^ab^	6.95 ± 4.84^a^	3.06 ± 2.55^b^
	Parasitic fungi	5.68 ± 3.59	2.37 ± 2.87	2.59 ± 3.62
	Endophytic fungi	0.22 ± 0.50	0.47 ± 1.13	0.14 ± 0.59
	Lichenised fungi	1.45 ± 1.23^a^	0.17 ± 0.28^ab^	0.05 ± 0.10^b^
	Epiphytic fungi	0.14 ± 0.81	0.05 ± 0.23	0.04 ± 0.15

**TABLE 2:** Linear model or Kruskal-Wallis* test statistics for significant differences in soil microbial alpha diversity metrics (richness and Shannon index), spatial turnover of taxa (distance decay index) and fungal guild relative abundances across old-growth forest (OG), naturally regenerating logged forest (NR) and actively restored logged forest (AR). Summaries are given for overall models and *post hoc* comparisons between forest types. *p*-values for pairwise tests for LMM and Kruskal-Wallis analyses were adjusted using the Tukey and Bonferroni methods, respectively. Significant p-values (> 0.05) are highlighted in bold. Sample numbers analysed were *n* = 168 for bacterial richness and Shannon diversity, *n* = 171 for fungal richness, Shannon diversity and fungal guild relative abundances, and *n* = 9 for both bacterial and fungal distance decay indices.

Metric	Soil microbial group	Overall model			Pairwise tests					
					OG - NR		OG - AR		NR - AR	
		*R* ^2^	*F*/χ^2^*	*p*	*t*-ratio/Z*	*p*	*t*-ratio/Z*	*p*	*t*-ratio/Z*	*p*
Richness	Total fungi	0.21	22.03	**<0.001**	6.37	**<0.001**	4.79	**<0.001**	−1.59	0.255
	Saprotrophic fungi	0.36	31.10	**0.001**	5.91	**0.003**	7.48	**0.001**	1.57	0.329
	Pathogenic fungi	0.21	7.58	**0.023**	3.69	**0.024**	2.93	0.060	−0.76	0.739
Shannon diversity index	Bacteria	0.21	8.71	**0.017**	−4.05	**0.016**	−2.89	0.063	1.16	0.514
	Total fungi	0.26	29.87	**<0.001**	7.56	**<0.001**	5.16	**<0.001**	−2.41	**0.045**
	Saprotrophic fungi	0.20	21.84	**<0.001**	−4.55	**<0.001**	−6.43	**<0.001**	−1.87	0.150
Distance decay index (rate of spatial turnover of taxa)	Bacteria	0.94	45.45	**<0.001**	9.23	**<0.001**	2.53	0.098	−6.70	**0.001**
	Mycorrhizal fungi	0.65	5.48	**0.044**	2.41	0.115	3.17	**0.044**	0.77	0.735
	Ectomycorrhizal fungi	0.77	10.15	**0.012**	3.51	**0.029**	4.20	**0.013**	0.69	0.777
Fungal guild relative abundance	Total pathogenic	0.28	12.64	**0.007**	2.44	0.110	5.03	**0.006**	2.59	0.091
	Plant pathogenic	0.30	14.06	**0.005**	4.63	**0.009**	4.55	**0.009**	−0.08	0.996
	Animal pathogenic	0.18	5.75	**0.040**	−0.60	0.824	2.59	0.091	3.19	**0.043**
	Lichenized fungal*	-	7.20	**0.027**	1.34	0.539	2.68	**0.022**	−1.34	0.539

**FIGURE 4:**
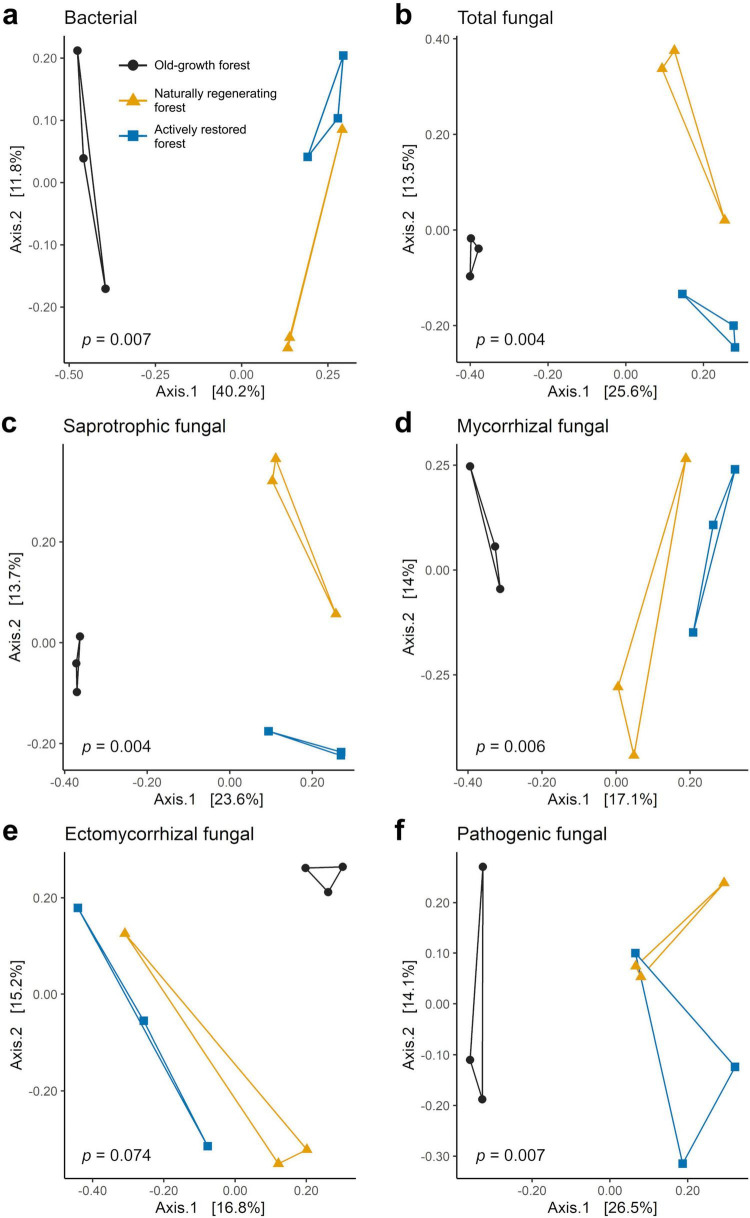
Principal coordinates analysis (PCoA) ordinations of Bray-Curtis dissimilarities for soil bacterial **(a)** and fungal **(b–f)** communities studied across old-growth forest (black), naturally regenerating logged forest (orange) and actively restored logged forest (blue) using data merged at the site level (*n* = 9). *p*-values denote significance of differences in community composition across forest types in PERMANOVA tests. See Table 3 for a full summary of statistical test results.

Bray-Curtis community dissimilarities were significantly affected by forest type for all soil microbial groups, with the exception of ectomycorrhizal fungi, which was marginally non-significant (Figure 4 and Table 3). Community dissimilarity dispersions were homogenous between all land-use types for all fungal groups (*p* > 0.05). UPGMA hierarchical clustering analysis (Supplementary Figure 4) identified communities in old-growth sites to be most dissimilar to logged forest sites across all microbial groups, with the exception of ectomycorrhizal fungi (bacteria: 86.52% dissimilarity; overall fungi: 89.84%; saprotrophic fungi: 87.46%; mycorrhizal fungi: 97.27%; pathogenic fungi: 78.91%). For ectomycorrhizal fungi, the greatest dissimilarity was found between OG-1 and all other sites (98.44%), followed by dissimilarity between OG-2 and OG-3 sites and all others (97.59% dissimilarity). Logged forest sites were generally more similar, with naturally regenerating and actively restored sites clustering together for all soil microbial groups.

**TABLE 3:** PERMANOVA test statistics for the effect of forest type on soil microbial community Bray-Curtis dissimilarities using data merged at the site level (*n* = 9). Significant *p*-values (< 0.05) are highlighted in bold.

Soil microbial group	*R* ^2^	*F*	*p*
Bacteria	0.49	2.89	**0.007**
Total fungi	0.38	1.85	**0.004**
Saprotrophic fungi	0.36	1.71	**0.004**
Mycorrhizal fungi	0.29	1.25	**0.006**
Ectomycorrhizal fungi	0.28	1.14	0.074
Pathogenic fungi	0.37	1.74	**0.007**

**FIGURE 5:**
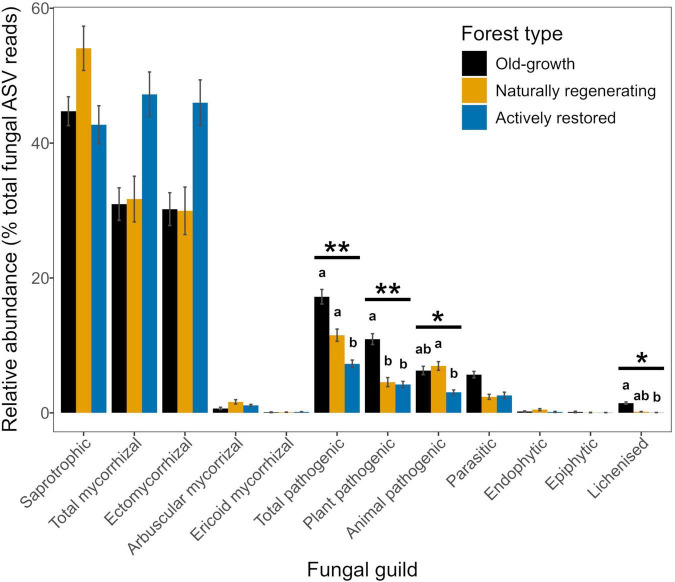
Relative abundances of fungal guilds (means ± 1 standard error) across old-growth forest (black), naturally regenerating logged forest (orange) and actively restored logged forest (blue) (*n* = 171). Horizontal bars and asterisks indicate significant overall effects of forest type on relative abundances of fungal guilds identified by linear mixed model or Kruskal-Wallis analysis, **p < 0.01, *p < 0.05. Lower case letters indicate statistically different or similar groups by forest type within fungal guilds at the *p* < 0.05 level identified in *post hoc* tests. See Table 2 for a summary of statistical test results. Means ± 1 standard error for fungal guilds with low relative abundances in old-growth forest (OG), naturally regenerating logged forest (NR) and actively restored logged forest (AR) are as follows: ericoid mycorrhizal fungi – OG: 0.09 ± 0.05, NR: 0.10 ± 0.03, AR: 0.14 ± 0.04; endophytic fungi - OG: 0.22 ± 0.07, NR: 0.47 ± 0.15, AR: 0.14 ± 0.08; epiphytic fungi - OG: 0.14 ± 0.11, NR: 0.05 ± 0.03, AR: 0.04 ± 0.02; lichenized fungi - OG: 1.45 ± 0.16, NR: 0.17 ± 0.04, AR: 0.05 ± 0.01.

Fungal guild relative abundances for total pathogens, plant pathogens, animal pathogens and lichens significantly differed by forest type (Figure 5 and Tables 1, 2). Total pathogenic and lichenized fungal relative abundances were significantly higher in old-growth relative to actively restored forest (naturally regenerating forest was similar to both old-growth and actively restored forest types for both fungal guilds), while plant pathogenic fungal relative abundance was significantly higher in old-growth relative to both logged forest types. Animal pathogenic fungal relative abundance was higher in naturally regenerating forest relative to actively restored forest (old-growth was similar to both logged forest types). PLFA analysis indicated no differences in total microbial, fungal or bacterial biomass or F:B between forest types (*p* > 0.05 in overall and pairwise tests).

### 3.2 Microbial indicators of forest types

Indicator analysis of bacterial taxa identified 566 significant indicator ASVs for old-growth forest, 99 for naturally regenerating forest and 139 for actively restored forest (Supplementary Data Sheet 2, see also for indicator ASV taxonomic classifications). At the phylum level, a larger proportion of indicator ASVs unique to old-growth appeared to belong to the Firmicutes in comparison to both logged forest types (Supplementary Figure 5a). Only two significant bacterial indicator ASVs were shared by old-growth and actively restored logged forest, both belonging to the phylum Firmicutes, class Bacilli (*Alicyclobacillus* sp. and *Bacillus foraminis*). 205 significant fungal indicator ASVs were identified for old-growth forest, 45 for naturally regenerating forest and 94 for actively restored forest. Notable differences in composition of fungal indicator taxa phyla was Mortierellomycota appearing to occur mostly only in old-growth forest, and Mucoromycota mostly only in actively restored forest (Supplementary Figure 5b). Four significant indicator ASVs were shared between old-growth and actively restored forest from the two phyla Ascomycota and Basidiomycota, and two classes Sordariomycetes and Tremellomycetes (*Trichoderma deliquescens*, *Clonostachys rosea*, *Castanediella* sp. and *Saitozyma podzolica*).

### 3.3 Soil physicochemical properties, microclimate and forest structural characteristics across old-growth, naturally regenerating and actively restored logged forest

Of all the soil physicochemical properties, microclimate and forest structural characteristics measured, only soil pH significantly differed between forest types (Table 4; overall model: *R*^2^ = 0.83, *F* = 50.56, *p* < 0.001). *Post hoc* tests identified significantly higher soil pH in old-growth forest relative to both logged forest types (*p* < 0.001), which did not significantly differ between themselves (*p* > 0.05). Mean understorey PAR was highest in naturally regenerating logged forest, although not significantly different due to high heterogeneity in this forest type.

**TABLE 4:** Soil physicochemical properties, microclimate and vegetation characteristics in old-growth forest, naturally regenerating logged forest and actively restored logged forest (means ± 1 standard deviation). Superscript letters indicate statistically different or similar groups at the *p* < 0.05 level identified in *post hoc* tests after linear mixed model analysis. Sample numbers analysed were *n* = 171 for soil and microclimate variables and *n* = 9 for forest structural characteristics (i.e., one value per site).

Group	Parameter	Forest type		
		Old-growth	Naturally regenerating	Actively restored
Soil	pH	5.57 ± 0.42^a^	4.02 ± 0.30^b^	3.89 ± 0.19^b^
	C (%)	5.87 ± 2.13	5.31 ± 1.67	4.25 ± 1.41
	N (%)	0.44 ± 0.14	0.40 ± 0.11	0.31 ± 0.07
	C: N ratio	13.23 ± 1.49	13.26 ± 1.23	13.52 ± 1.92
	Total P (μg g^–1^)	478.42 ± 142.44	253.39 ± 43.43	348.49 ± 89.32
	Inorganic P (μg g^–1^)	11.65 ± 7.63	14.55 ± 5.22	8.79 ± 4.23
Microclimate	Understory PAR (μmol m^–2^ s^–1^)	60.75 ± 83.99	144.37 ± 345.82	82.77 ± 194.71
	Soil temperature (°C)	26.29 ± 1.10	24.88 ± 0.66	26.37 ± 0.82
	Understory air temperature (°C)	26.20 ± 0.69	24.95 ± 0.44	25.86 ± 0.49
Forest structural	Basal area (m^–2^ ha^–1^)	93.55 ± 42.99	117.92 ± 25.11	141.36 ± 18.00
	Stem density (no. stems DBH ≥ 5 cm ha^–1^)	896.97 ± 98.93	1,336.99 ± 146.88	1,091.60 ± 235.66
	Mean stem diameter (cm)	26.23 ± 4.35	28.61 ± 1.73	27.21 ± 0.19

## 4 Discussion

We evaluated responses in soil microbial community attributes, soil physicochemical properties, microclimate and forest structural characteristics to active and passive restoration of logged Bornean lowland dipterocarp rainforest relative to old-growth forest across different spatial scales. Bacterial community attributes differed by forest type, including community composition (Figure 4a), alpha diversity metrics and rate of spatial turnover of taxa (Figures 3a, g, m), broadly supporting our first hypothesis. Specifically, results showed higher bacterial alpha diversity and lower rates of spatial turnover of taxa in naturally regenerating forest, in agreement with the observation that forest disturbance increases local bacterial diversity while homogenizing communities over larger spatial scales (Rodrigues et al., 2013). Although tropical forest disturbance is often accompanied by declines in both alpha and beta diversity of aboveground organisms (Bierregaard et al., 2001; Sodhi et al., 2009), this is not necessarily the case for belowground microbial communities where the opposite trend may be seen in local diversity patterns (Petersen et al., 2019). As discussed by Rodrigues et al. (2013), this “decoupling” of alpha and beta diversity with disturbance may depend on the relative effects of disturbance on ecosystem productivity. For example, alpha diversity in aboveground communities has been shown to increase with disturbance when productivity rates are higher in resulting ecosystems (Smart et al., 2006). In microbial terms, old-growth rainforests may be characterized by relatively low belowground productivity compared to adjacent open ecosystems created through anthropogenic disturbance (Cenciani et al., 2009; Cerri et al., 2004). In the current study system, increased bacterial alpha diversity with SL may potentially result from changes in vegetation characteristics, e.g., changes in tree community composition, or increasing understorey vegetation through creation of canopy gaps (Denslow, 1995) affecting the quality and quantity of plant inputs to the soil, improving effective resource availability (Cenciani et al., 2009; Cerri et al., 2004) and creating different ecological niches for bacterial communities (Zhang et al., 2018). This observation is congruent with the intermediate disturbance hypothesis, which predicts increases in diversity with a certain level of disturbance (Ferrenberg et al., 2013; Galand et al., 2016; Wilkinson, 1999; Zhang et al., 2011). While the measured soil properties were largely similar between forest types, soil pH, an important determinant of bacterial communities in Southeast Asian tropical forests (Tripathi et al., 2012), was significantly greater in old-growth forest sites (Table 4). The observed differences in soil bacterial community attributes disagree with some previous work in the same region of Borneo that found bacterial communities and diversity to be broadly resilient to selective logging (Lee-Cruz et al., 2013; Tripathi et al., 2016). This is likely due to the coarse spatial sampling resolution used in these studies to evaluate alpha and beta diversity (composite samples comprising soil collected up to 200 m apart). As bacterial community structure can vary considerably over meter-and centimeter-scales (O’ Brien et al., 2016), sampling resolution of previous surveys may be inappropriate for evaluating bacterial diversity and biogeographical patterns in response to forest ecosystem disturbance. The findings of the present study highlight the need for landscape studies of soil microbial diversity to incorporate fine spatial scale approaches to identify impacts and implications for biogeochemical cycling. Although clear differences in bacterial alpha diversity and spatial turnover of taxa were detected in naturally regenerating forest, findings suggest that restoration of selectively forest by enrichment planting can recover these metrics to levels comparable to old-growth forest. Bacterial alpha diversity may have potential as an indicator of rehabilitation of selectively logged forest, with lower values representing ecosystem recovery. This is opposite to trends in bacterial alpha diversity used to monitor progress of forest rehabilitation after total clearance, which can increase with replanting (Nurulita et al., 2016). This emphasizes the importance of disturbance history in identifying appropriate context-specific recovery indicators. Indicator analysis revealed old-growth forest bacterial communities harbor a large number of unique taxa that are not found in nearby logged forest, also evident for soil fungi (Supplementary Figure 5a, b), suggesting some aspects of ecosystem complexity are lost through disturbance and not recovered after almost two decades of active restoration or natural regeneration. A large proportion of unique bacterial taxa in old-growth forest belonged to the Firmicutes, a phylum associated with high soil C availability and resilience to environmental (microclimatic) perturbations (Battistuzzi and Hedges, 2009; Rodrigues et al., 2013), that were largely absent in unique taxa of both logged forests. While we found no differences in total soil carbon between forest types, greater abundance of unique taxa in this phylum may result from changes in the quality of plant-derived carbon inputs to the soil, e.g., logging disturbance has been linked to increased recalcitrance of tree litter carbon and reduced functional breadth of microbial decomposer communities relative to old-growth forest in the same area (Elias et al., 2020).

Some fungal community attributes also differed by forest type. While results broadly agree with observed logging disturbance effects of fungal communities (Kerfahi et al., 2014; McGuire et al., 2015; Robinson et al., 2020; Robinson et al., 2024), our results did not corroborate our prediction that overall fungal community composition would be more similar between old-growth and actively restored forest. Total, saprotrophic, and mycorrhizal fungal communities significantly differed by forest type, with old-growth forest communities appearing distinct from naturally regenerating and actively restored forest which were generally more similar (Figures 4b–f). Differences observed in the composition and rate of spatial turnover of mycorrhizal communities across forest types is consistent with studies showing high sensitivity of mycorrhizae to logging disturbance, likely due to the targeted extraction of ectomycorrhizal-associating dipterocarp trees (Kerfahi et al., 2014; Robinson et al., 2020; Robinson et al., 2024). The composition of mycorrhizal and ectomycorrhizal fungal communities in actively restored forest appeared even more distant to old-growth forest than naturally regenerating forest (Figures 4d, e), possibly reflecting differences in the mycorrhizal associations of tree species selected for planting during active restoration and those removed during timber extraction. Similarly, the rate of spatial turnover of mycorrhizal taxa was most different between old-growth and actively restored forest (the latter approximately three times lower), with rates in naturally regenerating forest similar to both (Figure 3p). Mean overall mycorrhizal and ectomycorrhizal richness, Shannon alpha diversity and relative abundances were also highest in actively restored forest, but did not significantly differ due to high variability (and likely resulting from low number of true spatial replicates in each forest type). There were surprisingly few fungal taxa shared between old-growth and actively restored forest (420 ASVs; Supplementary Figure 3b) relative to those shared between the two logged forest types (1,334 ASVs), or even old-growth and naturally regenerating forest (659 ASVs) – a pattern also evident in bacteria (384 shared between old-growth and actively restored forest; 2,843 between logged forest types; 1,047 between old-growth and naturally regenerating forest; Supplementary Figure 3a). This suggests that some taxa may be lost due to current restoration practices that are otherwise present in both old-growth and naturally regenerating forest. This is reflected in the lower total fungal alpha diversity in actively restored forest relative to old-growth (Figures 3b, h), which is likely driven by lower saprotrophic fungal alpha diversity (Figures 3c, i) as this guild represented the largest proportion of total fungal reads (47.17%). These findings may be potentially related to long-term control of liana species which have their own soil microbial associations (McGuire et al., 2008; Schnitzer et al., 2005), and removal of understorey vegetation which is practiced in these study sites. Vegetation removal may alter plant litter inputs, which in turn can affecting resulting microbial decomposer communities (Shi et al., 2019). Further study of the effects of liana and understorey vegetation removal through controlled field experiments (i.e., enrichment planting with and without additional vegetation clearance) is required to unpick the underlying drivers of these observations, and possible role in the impediment of fungal community recovery toward characteristics of old-growth forest.

No differences were found in indicators of overall microbial biomass between forest types, refuting our second hypothesis and contrasting with previous studies observing clear reductions in MBC in degraded forest relative to old-growth (Deng et al., 2010; Nurulita et al., 2016), or higher MBC in restored versus unrestored forest (Daljit Singh et al., 2013). In the present study, it is possible either microbial biomass was unaffected by logging in these forests, or returned to comparable levels with old-growth in both logged forest types with natural or managed regeneration during time since disturbance. The small number of samples used for analysis (*n* = 32) may also have contributed to lack of differences found due to a large amount of within- forest type (and site) variation.

Of all the soil physicochemical properties, microclimate and forest structural characteristics measured, only soil pH was affected by forest type (third hypothesis) with more acidic soils found in both logged forest types relative to old-growth (Table 4). Interestingly, bacterial alpha diversity was found to be higher in unrestored forest soils relative to old-growth despite lower pH, contrasting with studies across multiple biomes (including Malaysia) which found bacterial alpha diversity to increase with soil neutrality over land-use gradients (Lauber et al., 2009; Tripathi et al., 2012). Bacterial alpha diversity may therefore be influenced more strongly by altered litter inputs while soil microbial successional processes are still underway, rather than soil pH which may govern attributes of climax bacterial communities (also see Robinson et al., 2024).

## 5 Conclusion

In conclusion, while many soil properties and soil microbial attributes were similar across forest types, our results demonstrate contrasting responses of different soil microbial groups to active and passive restoration of selectively logged forest. Bacterial and fungal community composition remained generally more similar between logged forest types and more distinct in old-growth forest following 18 years of natural regeneration or enrichment planting. Bacterial alpha diversity and spatial turnover of bacterial taxa may recover toward old-growth forest levels with active restoration, while fungal alpha diversity showed slower signs of recovery largely due to saprotrophic fungal alpha diversity remaining lower in both logged forest types relative to old-growth forest. The composition and rate of spatial turnover in mycorrhizal communities was most different between old-growth forest and actively restored forest, possibly resulting from discrepancies in respective mycorrhizal associations of tree species planted during restoration and those removed during timber harvest. Few fungal taxa were shared between old-growth and actively restored forest, indicating recovery of soil microbial communities may be impeded by current management practices, with implications for carbon cycling. Further study into the effects of liana and understorey vegetation removal through controlled experimentation is required to test underlying mechanisms. Taken together our findings emphasize the importance of evaluating belowground microbial communities during forest restoration, particularly at fine spatial (cm to m) scales, to evaluate and predict recovery of biodiversity and ecosystem functions in human modified tropical forest.

## Data Availability

The molecular datasets generated for this study are available through the National Center for Biotechnology Information Sequence Read Archive (NCBI-SRA), Project no.: PRJNA1218937 (https://www.ncbi.nlm.nih.gov/bioproject/PRJNA1218937). The datasets of soil microbial community attributes (including ASV abundance tables, associated taxonomic and functional classifications, diversity indices, relative abundances of fungal guilds), soil properties, forest structural and microclimate parameters are available through the UKCEH Environmental Information Data Centre (EIDC) as part of the NERC Environmental Data Service (Robinson et al., 2025; https://doi.org/10.5285/08bfe302-d33f-490d-97be-27bb83a0f38d).
