# Supplementary material for: Soil microbial community responses to active and passive restoration of selectively logged Bornean tropical forest
Source: Front Microbiol. 2025 Aug 22;16:1570294. doi: 10.3389/fmicb.2025.1570294 (PMC12411923; doi:10.3389/fmicb.2025.1570294)
Supplement: Supplementary file 1 [file Data_Sheet_1.docx]

Supplementary Figures


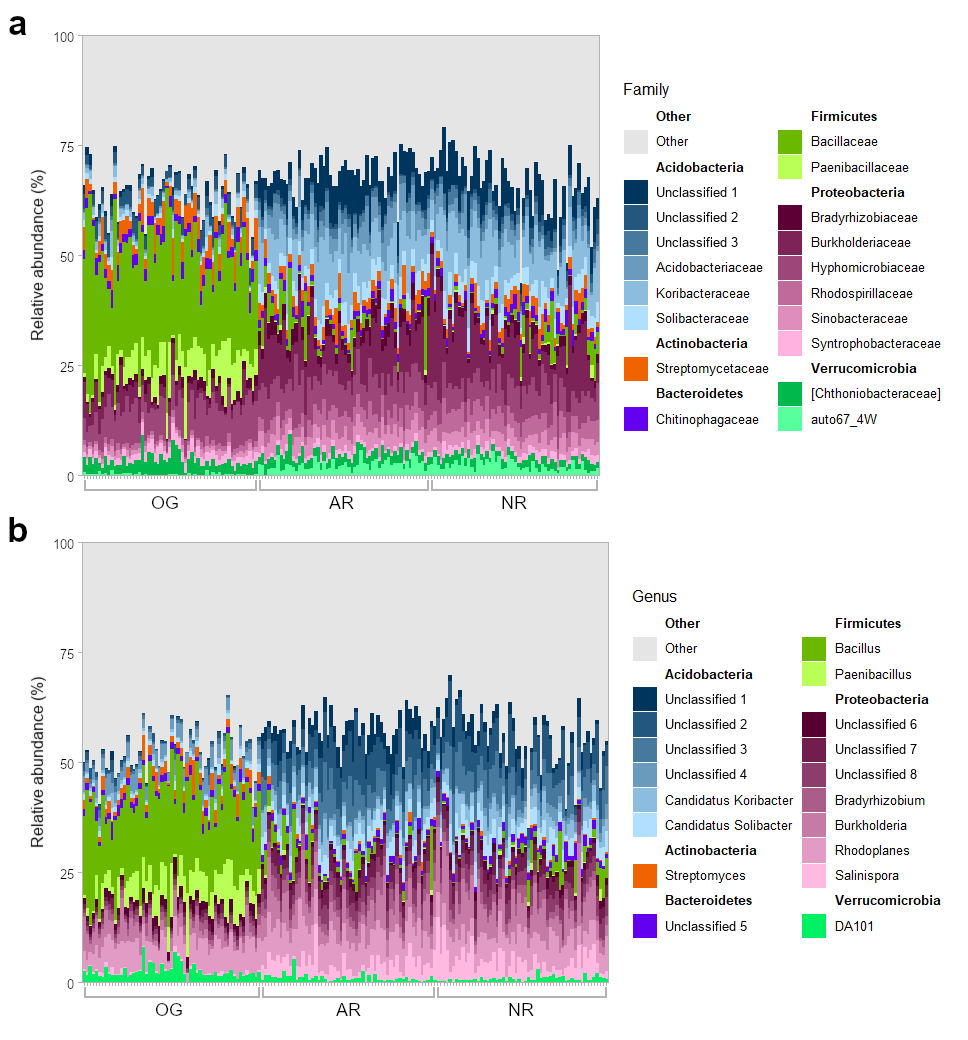


**Supplementary Figure 1.** Relative abundances of the top ten most abundant bacterial families (a) and genera (b) as percentages of total Amplicon Sequence Variants (ASVs) in old-growth forest (OG), actively restored logged forest (AR) and naturally regenerating logged forest (NR). Data are presented by individual sample (*n* = 168), grouped by forest type. Figure legends show family or genera nested within phyla (bold text), and distinct unclassified families and genera are indicated numerically.


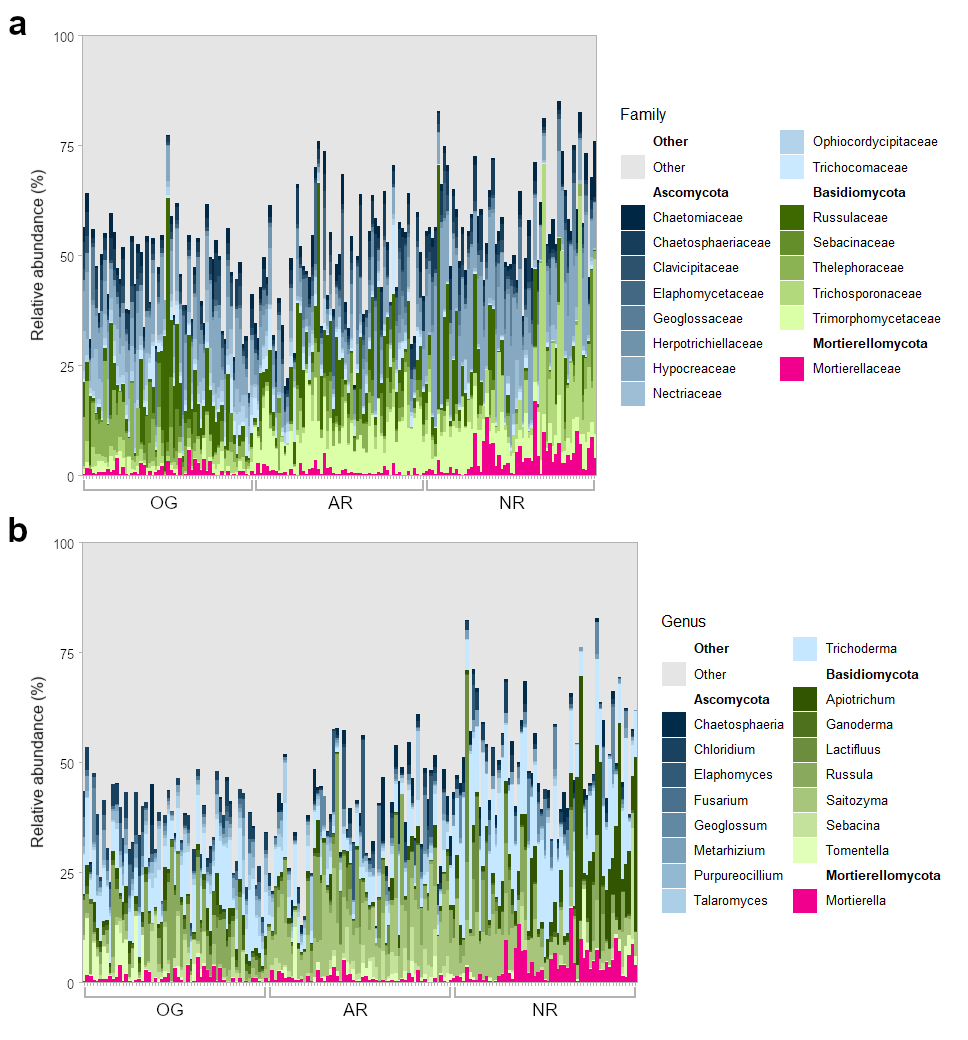


**Supplementary Figure 2.** Relative abundances of the top ten most abundant fungal families (a) and genera (b) as percentages of total Amplicon Sequence Variants (ASVs) in old-growth forest (OG), actively restored logged forest (AR) and naturally regenerating logged forest (NR). Data are presented by individual sample (*n* = 171), grouped by forest type. Figure legends show family or genera nested within phyla (bold text).

**
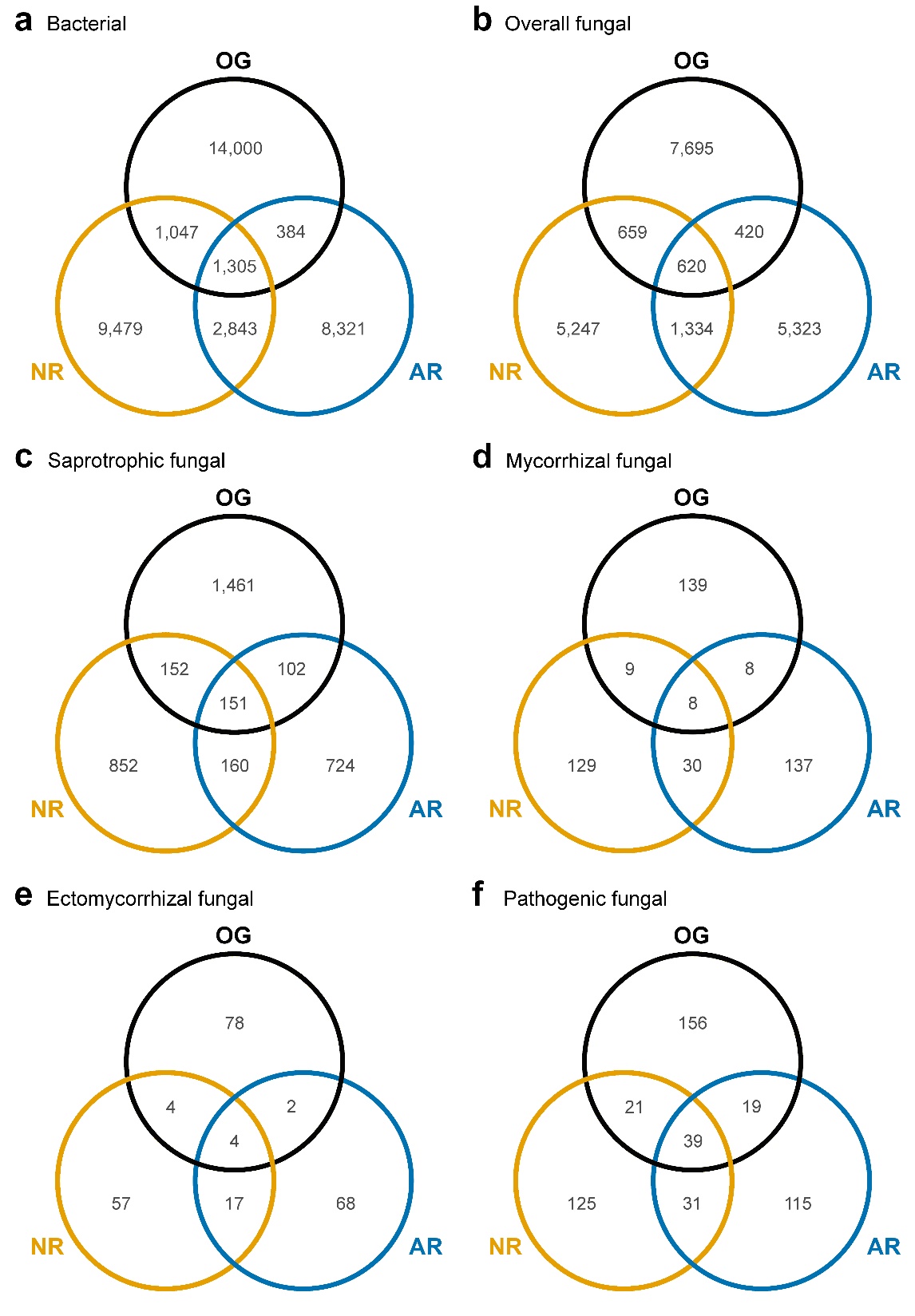
**

**Supplementary Figure 3.** Venn diagrams of numbers of Amplicon Sequence Variants (ASVs) unique to and shared between different forest types of old-growth forest (OG), naturally regenerating logged forest (NR) and actively restored logged forest (AR) for soil microbial groups studied (*n* = 168 and *n* = 171 for bacterial and fungal groups, respectively).
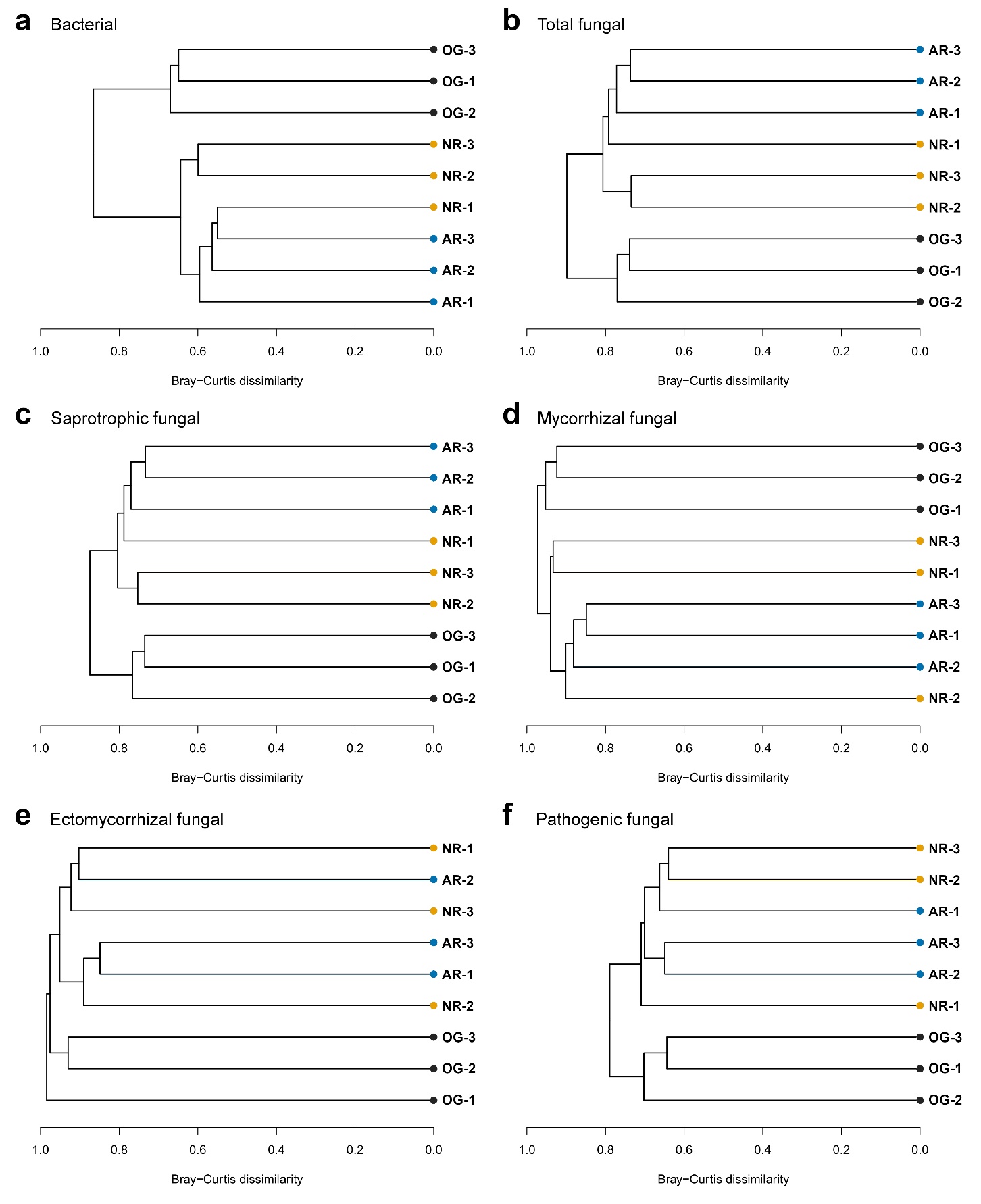


**Supplementary Figure 4.** Unweighted pair-group method with mathematic average (UPGMA) hierarchical cluster dendrograms illustrating Bray-Curtis dissimilarities between all sites across old-growth forest (OG; black), naturally regenerating logged forest (NR; orange) and actively restored logged forest (AR; blue) using data merged at the site level (*n* = 9).


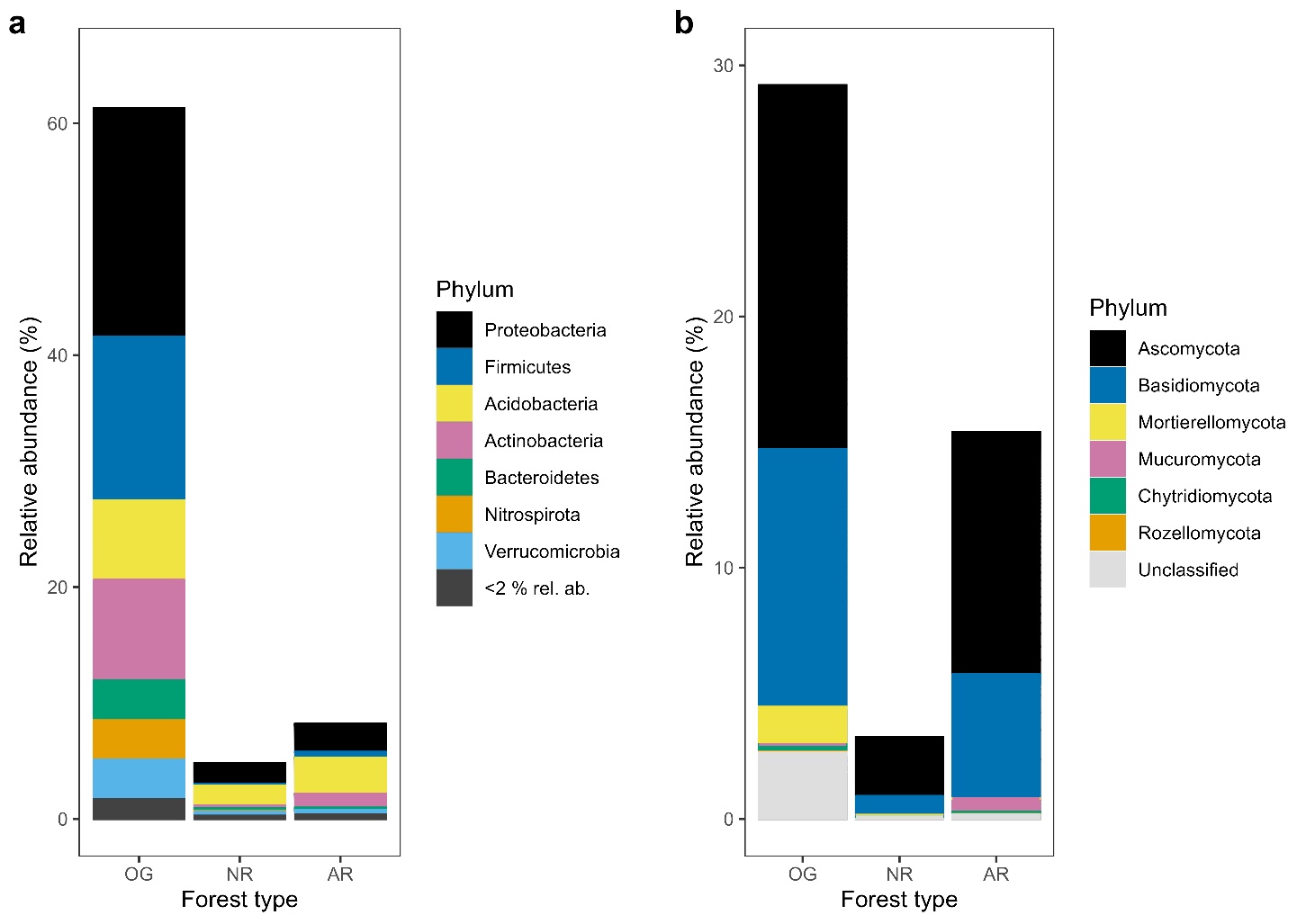


**Supplementary Figure 5.** Stacked bar graphs of relative abundances of bacterial (a) and fungal (b) indicator Amplicon Sequence Variants (ASVs) significantly unique to old-growth forest (OG), naturally regenerating logged forest (NR) and actively restored logged forest (AR) identified through indicator analysis using data merged at the site level (*n* = 9), grouped by phyla. Relative abundances are percentages of total ASVs. Bacterial phyla with < 2% relative abundance (rel. ab.) across all forest types are represented as one group.
